# Emergence of Order in Origin-of-Life Scenarios on Mineral Surfaces: Polyglycine Chains on Silica

**DOI:** 10.1021/acs.langmuir.2c02106

**Published:** 2022-12-05

**Authors:** Ola El Samrout, Marco Fabbiani, Gloria Berlier, Jean-François Lambert, Gianmario Martra

**Affiliations:** † Department of Chemistry, University of Torino, Via P. Giuria 7, 10125 Torino, Italy; ‡ Laboratoire de Réactivité de Surface, LRS, Sorbonne Université, Place Jussieu, 75005 Paris, France

## Abstract

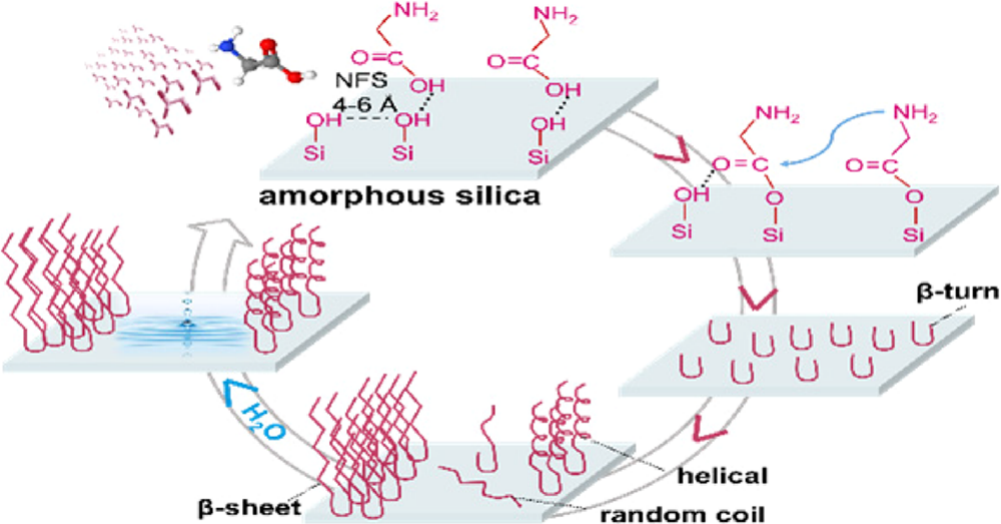

The polymerization of amino acids (AAs) to peptides on oxide surfaces has attracted interest owing to its high importance in biotechnology, prebiotic chemistry, and origin of life theories. However, its mechanism is still poorly understood. We tried to elucidate the reactivity of glycine (Gly) from the vapor phase on the surface of amorphous silica under controlled atmosphere at 160 °C. Infrared (IR) spectroscopy reveals that Gly functionalizes the silica surface through the formation of ester species, which represent, together with the weakly interacting silanols, crucial elements for monomers activation and polymerization. Once activated, β-turns start to form as initiators for the growth of long linear polypeptides (poly-Gly) chains, which elongate into ordered structures containing both β-sheet and helical conformations. The work also points to the role of water vapor in the formation of further self-assembled β-sheet structures that are highly resistant to hydrolysis.

## Introduction

The polymerization of amino acids (AAs) to peptides has attracted significant attention for a long time due to its importance in various fields ranging from biological applications,^1,2^ green synthesis development,^3,4^ to the study of the origin of life.^4,5^ A number of studies have suggested methods for polymerization in the absence of activating agents, such as the use of multiple wetting and drying cycles^6−8^ on bulk AAs. Peptide bond formation can also be accomplished by gently heating unactivated AAs at relatively low temperatures at the interface of inorganic oxide materials, such as silica or titania,^9−12^ a scenario that is considered of high interest in prebiotic chemistry. In his seminal work, Bernal^13^ proposed a key role for mineral surfaces in promoting peptide bond formation. In fact, their effect is twofold. Thermodynamically, they make polymerization favorable by allowing conditions of low water activity, and kinetically, they exhibit catalytic effects by increasing the reaction rate at a given temperature.^5,14^ The surface-catalyzed peptide bond formation is also of high relevance in several fields, such as bio/nanotechnology, drug delivery, and biomineralization.

Among all inorganic materials, silica is one of the most important and abundant minerals on earth’s crust, likely present in the primordial earth. According to the literature,^15^ most of the oldest rocks on the earth during Archean and Precambrian times are cherts (silica) or silicified. This suggests a high mobilization of silica even in the Hadean, where alkaline silica-rich seas and lakes most likely occur. The bare silica surface is characterized by two main chemical functionalities: silanol (Si–OH) groups and siloxane (Si–O–Si) bridges, whose distribution, nature, and density depend on the preparation method and thermal treatment and are directly responsible for the hydrophilic/hydrophobic character of the surface and its physicochemical behavior toward (bio)molecules.^16,17^ According to the literature, silica is highly suitable as a platform for oligomerization.^18^ However, the mechanism of peptide formation on silica is still poorly understood. One convenient way to study the mechanism of this reaction is to carry it out at the gas (amino acid vapor)/solid (silica surface) interface by chemical vapor deposition (CVD), where the influence of water may be minimized. In this setting, infrared (IR) spectroscopy under controlled atmosphere may be used as a characterization method. It is a powerful technique that provides useful and detailed information on the chemical transformation of the AAs during the reaction. In contrast, AA reactions are more difficult in the presence of water, which can initiate competing reactions in the system under study.^19^ Adsorbing amino acid from gas phase may be relevant for astrochemical scenarios; interestingly, the possible occurrence of polymerization reactions on the surface of space dust grains is suggested by the recent discovery of a protein analog (hemolithin, involving chains of glycine (Gly) and hydroxy-Gly residues) of extraterrestrial origin inside a meteorite.^20^

For years, Gly, the simplest amino acid, which can be formed in high quantities from gas-phase reactions and Miller–Urey-type experiments representing potential abiotic syntheses in diverse environments,^21^ has been used as a reference molecule to study AA/silica systems without the additional complexity introduced by the lateral substituent. This has led to a fruitful interplay between experimental^22,23^ and computational works^24−26^ dealing with AAs polymerization on mineral surfaces. Furthermore, recent studies have suggested that Gly may have been formed in presolar environments and/or inside meteorite parent bodies, which has made it extensively studied in astrochemistry as it could provide insights into the processes that took place prior, during and following the formation of the Solar System.^21^

One recent study published by Chien and Yu^27^ has highlighted the ester-mediated peptide formation as an efficient pathway for the formation of amino acid-enriched oligomers. Regarding the interaction of AA vapors with silica, early research^19^ suggested that ester linkages that may be formed during adsorption, also known as surface mixed anhydrides (SMA), play the role of “activated intermediates” in the oligomerization of AAs.^28^ Surface esters were originally thought to form through the esterification of the surface silanols by a carboxylic acid to form chemisorbed species on the surface.^29^ However, Rimola et al.^30^ established, in their computational work, that only highly strained two- or three-membered rings on the silica surface ((Si–O)_2_ and/or (Si–O)_3_) should be reactive toward a carboxylic moiety. Other surface groups that could play an important role in AAs oligomerization on a silica surface are silanol pairs. Rimola et al.^31^ recently suggested that the amide bond formation reaction specifically occurs at specific weakly interacting Si–OH pairs separated by a distance between 4 and 6 Å and known as nearly free silanols (NFS).

In the present work, we aim to (i) experimentally study the surface modifications during the adsorption and reaction of a carboxylic moiety on a silica surface to form ester species, (ii) investigate the role of ester species and NFS groups in the formation of linear poly-Gly produced by a continuous feeding of Gly from the vapor phase, and (iii) assess how the presence of these active sites on the surface affects the secondary structure and mobility of poly-Gly on amorphous silica.

## Experimental Section

### Materials

The commercial highly pure pyrogenic silica powder AEROSIL OX 50 (AOX50) (provided by Evonik, SiO_2_ content ≥ 99.8 wt %) with a specific surface area (SSA) of 50 m^2^ g^–1^ was used in the present work. Glycine (99%) from Sigma-Aldrich was used as received. Formic acid (FA) and deuterated water D_2_O (99.90 atom % D) were high-purity products obtained from Sigma-Aldrich. The vapors of these chemicals as well as those of Milli-Q water (Millipore system) were admitted onto the sample in the IR vacuum cell after several freeze–pump–thaw cycles.

### Pretreatment of the Silica Materials

Amorphous AOX50 silica has been selected for this work because according to Raman spectroscopy measurements reported in the literature,^32^ it contains a significant number of strained rings that could constitute reactive sites for the peptide formation reaction. In addition, its SSA of 50 m^2^ g^–1^ is high enough to obtain clearly detectable IR signals of surface species.^31^ Moreover, the used amorphous silica has been used in several works dealing with the abiotic polymerization of AAs^33,34^ and had been previously demonstrated to cause the formation of linear oligopeptides from AAs.^9^

A conventional IR cell for in situ measurements in transmission mode was used. This cell was equipped with a valve to connect it to vacuum lines (residual pressure 1 × 10^–5^ mbar) and composed of two main parts: one was dedicated to the thermal treatment and the other was an IR-transparent part with CaF_2_ windows for IR spectroscopic measurements. The temperature was measured by means of a thermocouple placed in contact with the external surface of the cell.

Silica AOX50 powder was pressed in the form of three self-supporting pellets denoted as AX_(rt)_, AX, and F-AX. The pressed SiO_2_ sample was put in a gold frame as a holder. The first two samples, AX_(rt)_ and AX, were just outgassed in the IR cell connected to a conventional vacuum line at room temperature (rt) or at 160 °C for 2 h (the latter treatment should allow to attain a complete surface dehydration before the start of glycine adsorption and polymerization reaction). These two SiO_2_ pellets were prepared for comparison with the formic acid-treated sample designated as F-AX.

To prepare the F-AX sample, a silica pellet was first outgassed at 160 °C for 2 h in the IR cell to attain a high dehydration level and to remove surface species, such as H_2_O, carbonates, etc. FA vapor (48 mbar) was admitted on the sample, which was then directly heated at 160 °C for 2 h in the closed IR cell. Subsequently, the pellet was again outgassed in the IR cell at beam temperature (bt) (ca. 50 °C) overnight. After that, water vapor (20 mbar) was admitted on the sample for 20 min before being outgassed for 30 min at bt and then for 2 h at 160 °C. This sequence of FA adsorption, heating at 160 °C, outgassing at bt, water contact, and then outgassing was repeated for three successive runs. After each successive step, an IR spectrum was measured in situ.

### Glycine Adsorption Procedure from the Gas Phase

For Gly sublimation and adsorption in situ in the IR cell, we used a similar method to Martra et al. based on CVD.^9^ In summary, after outgassing, the silica sample (AX_(rt)_, AX, or F-AX) was moved to the thermal treatment part for the start of the sublimation reaction where it was placed next to a Gly pellet and heated up to 160 °C in static vacuum for 2.5 h so that Gly started to sublimate and adsorb on the silica pellet. During this process, the valve connecting the cell to the vacuum line was closed and the cell was kept in contact with a liquid-nitrogen trap to remove water formed during Gly condensation reaction. After 2.5 h, the temperature was decreased to rt and the pellet was moved to the IR-transparent part for IR measurements. The sequence (contact with Gly vapor/IR spectra recording) was repeated until reaching 20 h of sublimation in total (steps of 2.5 h). The samples obtained after Gly adsorption were referred to as G/AX_(rt)_, G/AX, and G/F-AX.

The sublimation procedure was followed by (i) exposure to H_2_O vapor for 20 min with a subsequent outgas for 30 min at bt and (ii) H/D exchange by exposure to D_2_O vapor with a subsequent outgas for 30 min at bt. The D_2_O adsorption/desorption cycle was repeated until invariance of the IR spectra was observed.

### Analysis of the Products Extracted by Washing and of the Washed Samples

After the experiments of contact with Gly vapor followed by exposure to H_2_O vapor and H/D exchange were performed on the three different samples (AX_(rt)_, AX, and F-AX), the pellets were removed from the IR cell and each of them was ground manually in an agate mortar before suspending it in 0.5 mL of Milli-Q water. The three suspensions were shaken for 10 min by a Vortex mixer then centrifuged at 10,000 rpm for 10 min. For each sample, the supernatant was recovered and the solid was extracted four more times using the same volume of water. The first two aliquots of the aqueous solutions were mixed and analyzed by high-resolution mass spectrometry (HR-MS).

As regards the solid phase, the washed sample of G/F-AX was dried under a flow of nitrogen then pelletized and put in an IR cell for subsequent measurements to observe any organic matter remaining on the surface. First, the sample was outgassed at bt before the admission of D_2_O vapor in the vacuum line for several adsorption/desorption cycles until invariance of IR spectra.

### IR Spectroscopy

Throughout the treatment procedure, the pellets were monitored by means of in situ IR spectroscopy. The spectra were collected with a Bruker VECTOR22 instrument (resolution 4 cm^–1^, DTGS detector) at bt (∼50 °C) by accumulating 64 scans to obtain a good signal to noise ratio. The spectra were reported in the absorbance mode after scattering correction.

Integrated areas of the amide I band were calculated with the OPUS software (Bruker Optics GmbH) using the Levenberg–Marquardt algorithm. The peak fitting was performed using Gaussian and Lorentzian function.

### HR-MS Analysis

The washing solutions obtained were analyzed by HR-MS using an LTQ Orbitrap mass spectrometer (Thermo Scientific) equipped with an atmospheric pressure interface and an electrospray ionization (ESI) source. The source voltage was set to 4.48 kV. The heated capillary temperature was maintained at 265 °C. The tuning parameters adopted for the ESI source were as follow: capillary voltage 0.02 V, tube lens 24.77 V, for ions optics: multipole zero offset −4.28 V, lens zero voltage −4.36 V, multipole zero offset −4.28 V, lens 1 voltage −13.69, gate lens voltage −8.84 V, multipole 1 offset −18.69 V, and front lens voltage −5.09 V. The mass accuracy of recorded ions (vs calculated) was ±1 mmu (without internal calibration). The samples, added to 100 μL of a 0.1 M HCOOH aqueous solution, were delivered directly to mass spectrometer via Hamilton microliter syringe at a constant flow (10 μL/min).

### X-ray Diffraction (XRD)

The samples were characterized by X-ray powder diffraction patterns recorded on a PANalytical X’Pert diffractometer using a Cu Kα (λ = 1.5405 Å) radiation source and working at 30 mA and 40 kV. The diffractograms were recorded for 2θ angles ranging from 10° to 45° with a step size of 0.01° and a dwell time of 1 s per step.

### Thermogravimetric Analysis (TGA)

TGA of crushed pellets was carried out using a TA instrument with a STD Q600 analyzer. TGAs were performed with a heating rate of 1 °C/min under dry air flow (100 mL/min). Quantification of adsorbed peptides was evaluated by correcting the weight loss between 130 and 400 °C for the corresponding value for the blank sample.

## Results and Discussion

Amorphous AEROSIL OX 50 SiO_2_ powder has been used as it was selected in several previous studies dealing with amide/peptide bond formation.^9,31^ In these studies, Gly sublimation temperature was optimized at 160 °C and therefore, this temperature was selected to pretreat the silica sample under vacuum (this silica sample is hereafter labeled as AX after this treatment) and in the presence of FA (labeled as F-AX; IR spectra recorded after the pretreatment of silica samples are shown in Figure S1 in the Supporting Information).

In situ IR spectroscopy under controlled atmosphere was used to follow the surface modifications during the adsorption and reaction of FA on silica and during the successive steps of Gly deposition (adsorption and polymerization) on the two pretreated silica samples (hereafter noted as G/AX and G/F-AX).

### Adsorption and Reaction of FA on the Silica Surface at 160 °C

FA, the simplest carboxylic acid, was adsorbed from vapor phase on the silica surface pretreated at 160 °C (AX). FA vapor (48 mbar) was dosed at rt on AX, which was then heated at 160 °C for 2 h, still in the presence of the acid and finally outgassed overnight (residual pressure 10^–4^ mbar) at IR bt.

**Figure 1:**
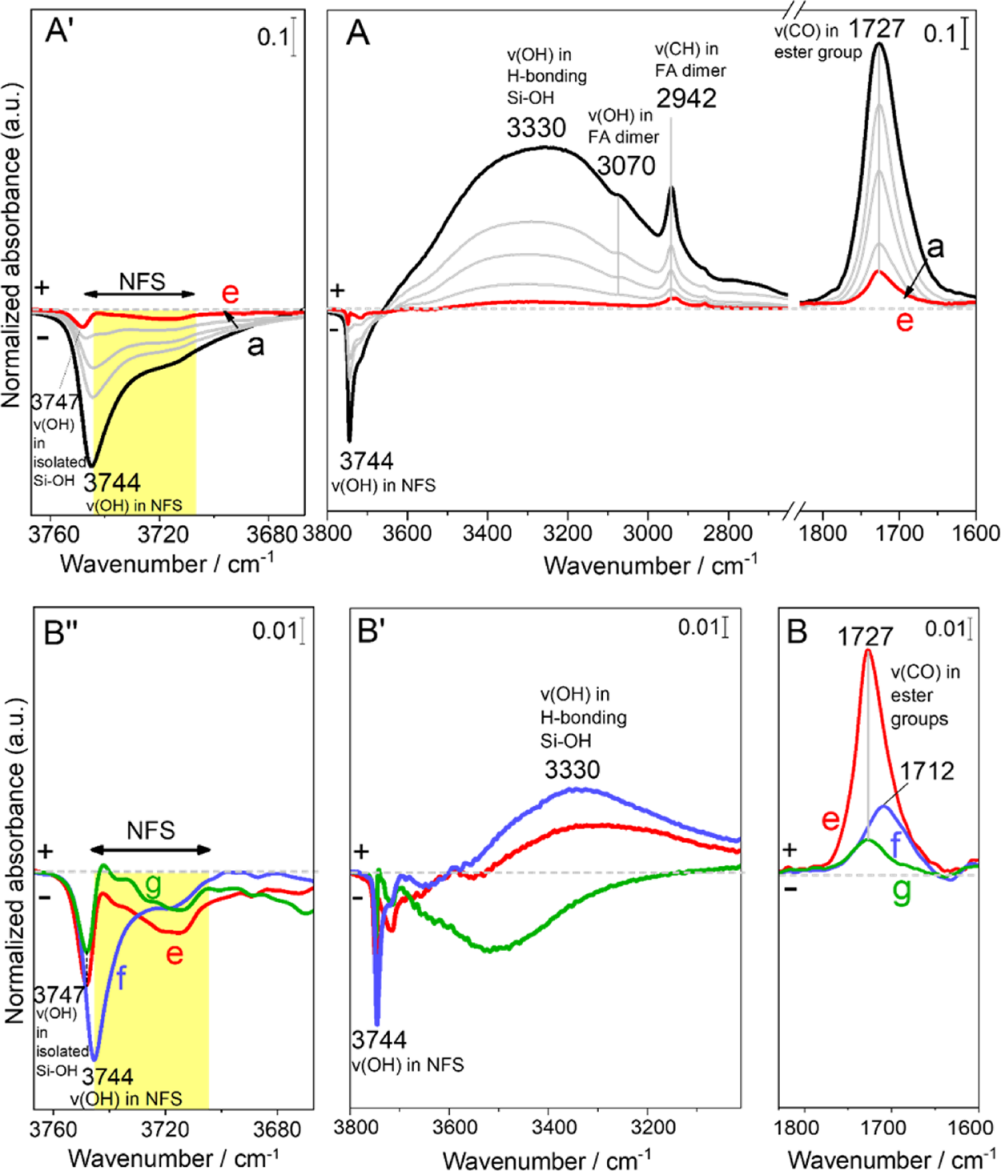
IR spectra of F-AX for the first run: (a) after treatment in FA vapor (48 mbar) at 160 °C for 2 h; (a–e) after outgassing overnight at bt until invariance of spectra; (f) after contact with water vapor (20 mbar) for 30 min followed by outgassing at bt; and (g) after outgassing at 160 °C for 2 h. The spectrum of bare SiO_2_ after outgassing at 160 °C for 2 h (AX) is subtracted as a baseline. In panels B, B′, and B″, the intensities are enhanced for the sake of clarity.

Figure 1A shows the difference spectra during the outgassing of the first adsorption cycle (curves a–e) of the sample F-AX. At low wavenumbers, the IR profiles show the progressive decrease in intensity of the band at 1727 cm^–1^ attributed to the ν_CO_ of FA. At high wavenumbers, a signal at 2942 cm^–1^ and a shoulder at 3070 cm^–1^ are assigned to the ν_CH_ and ν_OH_ of the same molecule. The ν_CH_ and ν_OH_ bands are close to their position in FA dimers according to the literature, while the ν_CO_ is significantly redshifted.^35^ After prolonged outgassing (curve e), the ν_CO_ signal is conserved in contrast to what was previously reported for FA adsorbed at rt (i.e., without thermal treatment) on the same silica surface.^31^ Its redshift as compared to the vapor phase forms (about 15 cm^–1^ with respect to the dimer and 39 cm^–1^ with respect to the monomer)^35^ suggests the occurrence of a strong interaction with the silica surface. The overall behavior of the IR signals upon outgassing is compatible with the fact that the treatment at 160 °C has favored the formation of strongly bonded surface ester species Si_surf_–O–C(=O)–H as observed in the case of acetic acid.^29^ In addition, no evidence of the formation of formate was detected on the surface^15^ excluding a strong electrostatic interaction.

In the ν_OH_ region, the silanol pattern is affected by the presence of FA molecules on the surface. During outgassing (curves a–e), the progressive decrease of the broad positive band centered at 3330 cm^–1^ (curve a) attributed to H-bonded silanols is accompanied by the recovery of the negative profile peaking at 3744 cm^–1^, where weakly interacting silanols are found.

Even after long outgassing (Figure 1′A, curve e), some of the intensity of the band of weakly interacting silanols is not fully restored. Noticeable residual intensities are left in the region of isolated (3747 cm^–1^) and NFS (3744–3742 cm^–1^).^36^ This suggests that resilient ester species (with ν_CO_ around 1727 cm^–1^) were formed through a reaction with this type of silanols.

At the end of the adsorption/desorption cycle, water vapor (20 mbar) is contacted with the sample and outgassed at bt prior to a final outgas at 160 °C for 2 h and the spectra at the end of each outgas are being shown in Figure 1′B,B.

After contact with water vapor and rt outgassing (curve f), ν_CO_ undergoes an important decrease in intensity leaving a signal at 1712 cm^–1^ of residual adsorbed FA molecules. Contact with water probably promotes the hydrolysis of a large fraction of the surface ester species, but since outgassing is performed at bt, the FA molecules remain H-bonded with a ν_CO_ at 1712 cm^–1^; they are probably H-bonded to NFS groups (strong negative signal at 3744 cm^–1^ and positive signal for corresponding H-bonded silanols at 3300 cm^–1^).

After the final outgassing at 160 °C (curve g), only the underlying signal of the nonhydrolyzed ester species is left (apparently those that were formed on terminal silanols), while the NFS previously H-bonded to FA are restored. This high temperature outgassing removes the residual FA molecules leaving only some leftover surface esters at 1727 cm^–1^. The preferential recovery of the NFS (Figure 1′B) indicates that indeed on these species, FA can react and be hydrolyzed depending on the chemical environment it is interacting with.

The described sequence of FA adsorption, heating at 160 °C, outgassing at bt, and water contact then outgassing was repeated for three successive runs in the hope to increase the amount of surface esters (corresponding IR spectra in the ν_CO_ region reported in Figure S2).

### Gly Deposition and Polymerization on Silica Surfaces in CVD Conditions

**Figure 2:**
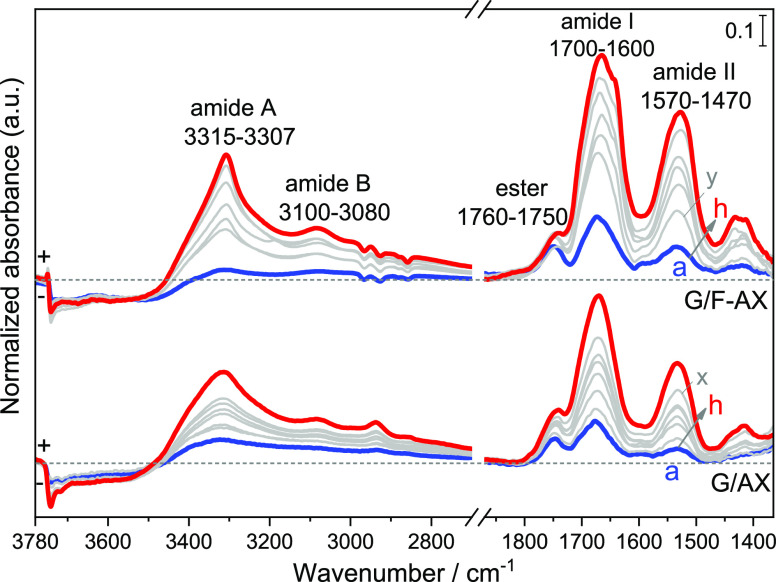
IR difference spectra resulting from Gly sublimation by CVD at 160 °C measured from 2.5 h (a) to 20 h (h) (gray curves show intermediate sublimation steps of 2.5 h) on the two samples: G/AX and G/F-AX. The corresponding spectra of the materials obtained before the start of CVD process are subtracted as baselines.

Gly vapor was then adsorbed at 160 °C for 20 h by CVD on AX and F-AX following the procedure adopted by Martra et al.^9^Figure 2 shows the IR spectra recorded after 20 h CVD (with steps of 2.5 h) for both samples, named G/AX and G/F-AX.

At low frequency, the IR profiles of both samples show a progressive increase in intensity of the bands formed in the 1700–1600 and 1570–1470 cm^–1^ ranges (curves a–h) identified as amide I and amide II, respectively.^37^ These bands exhibit higher intensities for G/F-AX for each step until 20 h CVD (curve h for each sample). The presence of the amide II band is characteristic of the formation of linear peptides rather than of cyclic products.^9^ In addition, the appearance of a band in the 1760–1750 cm^–1^ range indicates the formation of surface ester groups involving Gly molecules [Si_surf_–O–C(=O)–CH_2_–NH–R].^9,19,30^ Their behavior will be further discussed in the comments to Figure 5.

At high frequency, the IR profiles for both samples display bands in the 3315–3307 and 3080–3100 cm^–1^ ranges, which correspond to ν_NH_ in poly-Gly species, designated as amide A and B, respectively. They progressively increase in intensity and narrow in shape with increasing contact times and are more intense and narrower for G/F-AX than for G/AX after 20 h CVD. This is an indication of the formation of peptides with more ordered self-assembled structures on G/F-AX.^9^ The XRD pattern of the final samples (G/AX and G/F-AX, Figure S3 in SI) indicates that after 20 h CVD, no crystalline glycine (or crystalline peptides) is present in the sample: we are exclusively dealing with adsorbed species.

The relative amount of peptides may be evaluated from the integrated area of the amide I band (Figure S4 in SI). For all three samples (G/F-AX, G/AX, and G/AX_(rt)_), the temporal evolution of peptide bands can be roughly fitted with straight lines with nonzero intercepts. This would be compatible with the fast initial formation of small linear chains that elongate with a constant growth rate. On G/F-AX, peptides are significantly more abundant than on G/AX for the same time of Gly sublimation with G/AX_(rt)_ showing the smallest amounts. Thus, the FA modified sample is the most efficient platform for peptide formation and growth.

Quantifying the absolute amount of surface peptides from IR is more difficult. Yet, TGA data (Figure S5 in SI) on the final pellet (taking into account the desorption efficiency and vide infra) indicate that the maximum amount of peptides after 20 h CVD is about 3.25% by weight with respect to the silica support. This value would translate to a density of Gly residues of 6.9 per nm^2^. In Figure S3, this value was used as a basis to provide a *y*-axis graduated in units of Gly residues density supposing that the extinction coefficient of poly-Gly species remained constant.

**Figure 3:**
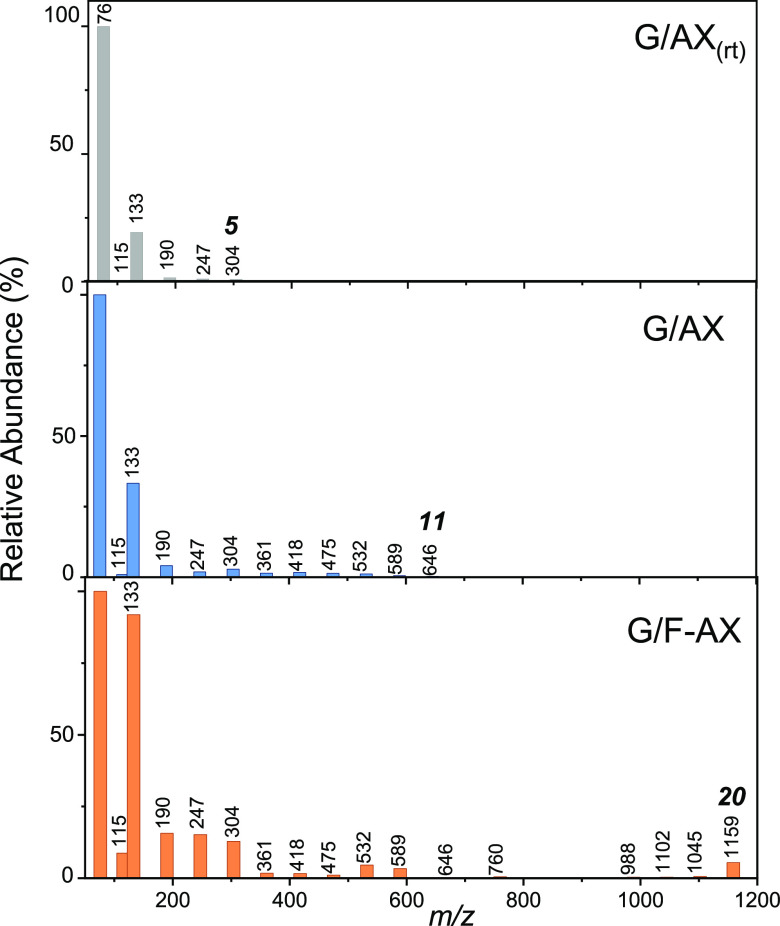
HR-MS spectra of the solutions resulting from washing (with pure water) of the samples produced by adsorbing Gly from the vapor phase onto the three samples: G/AX_(rt)_, G/AX, and G/F-AX. Numbers on the black bars are the *m*/*z* values of singly protonated species derived from (−Gly−)_*n*_ peptides. The number of monomers present in the peptides detected on the samples is indicated in italics above the corresponding signal.

HR-MS analysis of the peptides desorbed from silica surface by washing with ultrapure water (Figure 3) reveals the formation of longer poly-Gly chains containing at least 20 (*m*/*z* = 1159 for (Gly)_20_H^+^) monomers for G/F-AX compared to oligomers up to 11 (*m*/*z* = 646) monomers units formed on G/AX coherent with what was reported by Martra et al.^9^ for a similar sample and only five (*m*/*z* = 304) monomers for G/AX_(rt)_. Thus, the longer (desorbed and solubilized) chains are observed for samples that showed the more organized peptides based on the amide A and B bands. It must be underlined that washing with water only allows solubilization of ca. 24% of the formed peptides (see later), indicating that a considerable fraction of the Gly polymerization products are strongly bonded to the surface likely through the surface ester group in [Si_surf_–O–C(=O)–CH_2_–NH–R] (band at 1760–1750 cm^–1^). Thus, not much can be deduced from the high intensity of the Gly monomer and Gly–Gly dimer signals: these species may just be the easiest to desorb. The weak signal of the cyclic dimer (*m*/*z* = 115) with respect to the linear dimer is worth mentioning, however. It confirms that contrary to what is often observed after aqueous phase deposition, the cyclic dimers are not a predominant product.

**Figure 4:**
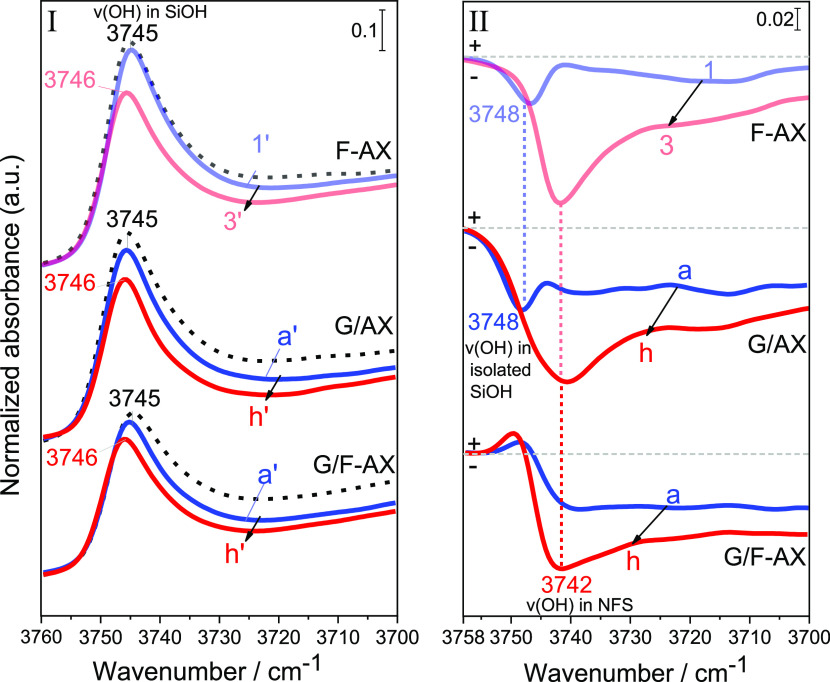
Enlarged sections in the 3760–3700 cm^–1^ range of IR spectra measured on F-AX, G/AX, and G/F-AX. Panel I shows the direct IR spectra of Si–OH populations present on the surface after the first and last step of contact with (1′ and 3′) FA or (a′ and h′) Gly. The dotted spectra refer to the corresponding material before any reactant contact (AX for the first two sets and F-AX for the last one). Panel II shows the corresponding difference IR spectra obtained using the corresponding spectra before contact with the desired reactant (dotted curves in panel I) as baselines.

When focusing on the silanol region (Figure 4), it appears that the first silanols to disappear upon contact of a pristine surface with either FA (F-AX) or Gly (G/AX) were the isolated terminal silanols as witnessed by a negative signal at 3748 cm^–1^. Later on, the NFS are also affected (negative signal at 3742 cm^–1^). In contrast, when Gly was deposited on the FA pretreated sample (G/F-AX), only the NFS groups were affected even at the start of the deposition. This could be expected since in this sample, isolated terminal silanols are apparently already esterified with FA even after hydrolysis and outgassing (cf. discussion of Figure 1). Thus, the behavior of G/AX is different from that of G/F-AX in the first deposition steps, but the two become similar at later CVD steps.

A similar behavior between the last CVD steps in G/AX and the first steps in G/F-AX is also evident in the evolution of the amide A, amide I, and amide II bands (compare curve *x* and curve *y* in Figure 2). This suggests that the reaction of the carboxylic group of Gly on AX at 160 °C modifies the silica surface in the same way as the carboxylic group of FA does. In other words, Gly dosed on silica by CVD does not only act as a reactant for polymerization but also as a surface modifier in forming surface esters.

**Figure 5:**
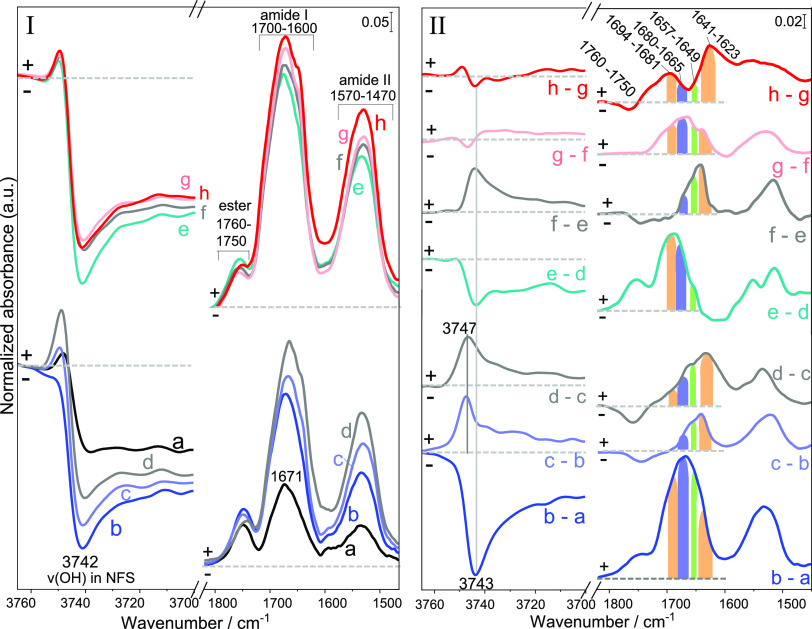
Enlarged sections of IR spectra during Gly sublimation by CVD at 160 °C for 20 h (in 2.5 h steps, from 2.5 h (a) to 20 h (h) sublimation) on G/F-AX. Panel I shows the 3760–3700 cm^–1^ (containing Si–OH stretching vibrations) and the 1820–1450 cm^–1^ ranges (containing the ester, amide I, and amide II vibrations) using the spectrum before contact with Gly as baseline correction. Panel II shows the double difference IR spectra in the 3760–3700 cm^–1^ and in the 1820–1450 cm^–1^ ranges (difference between each step and the previous one). The colored bars refer to the expected ranges for amide I in β-turns conformations (blue), helices (green), and β-sheets (orange).

A more in-depth analysis of the behavior of G/F-AX during Gly polymerization (Figure 5, panel I) allows to observe a negative correlation between the evolution of NFS groups (negative peak at 3742 cm^–1^) and that of the ester groups (positive peak at 1750–1760 cm^–1^) throughout the 20 h CVD, while the peptide bands (amide I and II) are progressively increasing. To better appreciate the trend, we plotted the double difference spectra between successive CVD steps (Figure 5, panel II). In all these spectra, a negative NFS signal corresponds to a positive ester one and vice versa. This suggests that NFS are specifically converted to esters, and conversely that the destruction of esters may regenerate NFS (and isolated silanols especially in the first CVD steps).

### Self-Assembly and Secondary Structures of Poly-Gly

Further information about the growth and evolution of polypeptides on the silica surface can be obtained by a closer analysis of the amide I band for G/F-AX, whose position strongly depends on the secondary structure of the peptides. After the first 2.5 h CVD (curve a, Figure 5, panel I), the amide I band is symmetrical and peaks at 1671 cm^–1^. This falls in the typical range of β-turns conformations (1680–1665 cm^–1^) and definitely outside the ranges of helices (1657–1649 cm^–1^) and β-sheets (1694–1681 and 1641–1623 cm^–1^ ranges) and even of disordered structures (1642–1647 cm^–1^).^38−41^ Starting from the following 2.5 h CVD step (curve b, Figure 5, panel I), a significant increase in the intensity of amide I and II bands is detected, suggesting that poly-Gly chains progressively become long enough to exhibit structure as the reaction proceeds. The evolution of their secondary structures along 20 h CVD is detected from the change in shape of the amide I band in the double difference spectra (Figure 5, panel II); the components of this band may be identified from the computation of the second derivative of the spectra (Figure S6). From 5 h till 20 h CVD, poly-Gly chains containing β-sheets and helices are formed in different quantities besides β-turns conformations. Some nonordered structures are also formed during some intermediate CVD steps on the basis of a minimum at 1642 cm^–1^ in the corresponding second derivative (Figure S6 in SI). In parallel, the narrowing of the ν_NH_ band (“amide A” in Figure 2) in the spectra of G/F-AX starting from 5 h CVD constitutes additional evidence of the formation of ordered structures.^9^

**Scheme 1:**
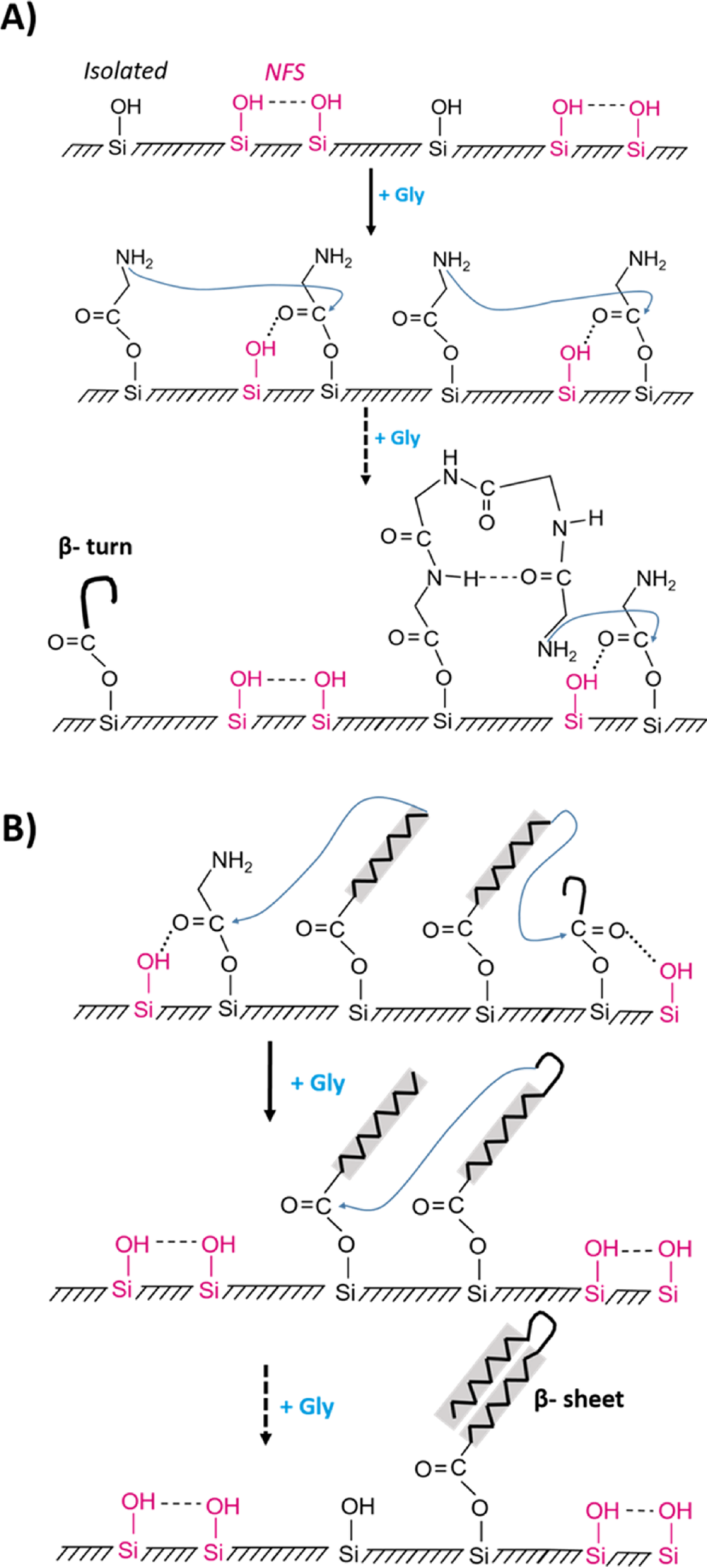
Suggested Scheme for (A) β-Turn and (B) Ligation and β-Sheet Structures Formation

During the first 5 h of CVD, the formation of a significant amount of β-turns is detected. They are probably grafted on the surface by ester groups (positive band in 1750–1760 cm^–1^ range, Figure 5, panel II, curve b–a). Each of these ester species would be formed by reacting with one silanol of the NFS pair (negative band pointed at 3743 cm^–1^) and probably stabilized by hydrogen bonding with the second silanol of the NFS (Scheme 1A) although on G/AX additional adsorption on isolated silanols may occur. This may appear in contradiction with calculations by Rimola et al. showing that ester formation between Gly and silanol is endergonic;^25^ however, this reaction is a condensation implying water elimination and in conditions of low water activity, it may still be possible.

The β-turn configuration that predominates in the initial stage involves four Gly monomers and can be stabilized by its terminal −NH_2_ group pointing toward the silica surface, and thus allowing H-bond to silanols (Scheme 1A).^42^

As regards the mechanism of the peptide bond condensation, esterification of the Gly carboxylic terminus is expected to decrease the electron density on the ester carbon atom favoring a nucleophilic attack from the amine terminus of another Gly molecule although the complete story probably also involves participation of neighboring H-bonded silanols (Scheme 1A).^30^ Another difficulty is that if the mechanism involved a Gly coming from the gas phase, it should result in desorption of the Gly residue, or of the polyglycine chain, that was initially bound to the surface as an ester.

We suggest that after a certain threshold of ester density on the surface, the grafted chains start to interact with each other (curves c–b, d–c, Figure 5, panel II); then, the terminal amino group of one surface-linked chain attacks the activated ester function of another poly-Gly chain (Scheme 1B).^43^ This results in the destruction of some esters and regeneration of the corresponding NFS and isolated silanols (negative band in 1750–1760 cm^–1^ range and positive one at 3743 and 3747 cm^–1^). The substantial chain growth entailed by these condensations results in the formation of β-sheets conformations as major elements. After this process that might be called “ligation,” the regenerated NFS can form ester groups with gas-phase Gly again then giving rise to new β-turn chains (curve e–d, Figure 5, panel II). At later stages (curves g–f and h–g, Figure 5, panel II), the surface is largely occupied by ordered structures that continue to elongate under continuous feeding of Gly monomers in keeping with the progressive increase in intensities of amide I and II until 20 h CVD (Figure 5, panel I).

Finally, one can wonder why FA pretreatment causes the surface to accumulate more poly-Gly chains. If, as we suggested, NFS groups play an important role in chain growth, it could mean that FA treatment creates more NFS. This could indeed be the case because FA could react with constrained siloxane rings yielding after hydrolysis pairs of silanols that would be in the NFS range.^30^

### Effect of Hydration/Dehydration Cycles on Grafted Poly-Gly

**Figure 6:**
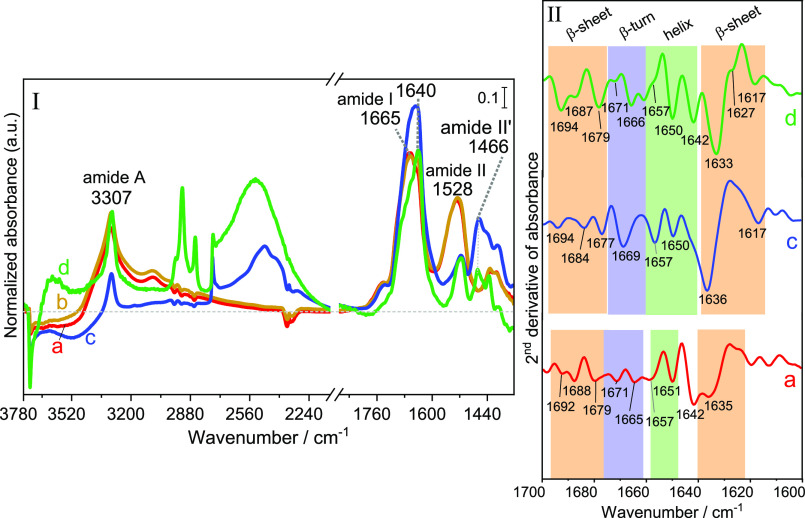
Panel I: IR spectra of G/F-AX submitted to successive treatments: (a) directly after Gly sublimation for 20 h, (b) after subsequent contact with water vapor (20 mbar) and outgassing for 30 min at bt, (c) after H/D exchange and then outgassing of D_2_O for 30 min at bt, and (d) after sample washing with ultrapure water followed by H/D exchange (then bt outgassing). The spectrum of the material obtained before the start of the CVD process is subtracted as a baseline. Panel II shows the second derivative of the IR spectra (a, c, and d) in the amide I region.

Since Martra et al. have reported that poly-Gly rearrange on the TiO_2_ surface to form self-assembled aggregates when contacted with water vapor,^9^ G/F-AX (Figure 6) and G/AX (Figure S7) were exposed to water vapor (20 mbar) directly after the end of 20 h CVD to study the events that occur upon hydration. For G/F-AX, the IR profile collected after outgassing the excess of water vapor (Figure 6, panel I, curve b) shows a slight narrowing in the ν_NH_ band of poly-Gly in the 3315–3307 cm^–1^ range and the appearance of a component at 1640 cm^–1^ in the region of the amide I band. The change in the ν_NH_ band upon hydration is more significant on G/AX (Figure S7) that initially (before exposure to water vapor) showed less ordered structures than G/FA-X.

Subsequently, D_2_O adsorption/desorption cycles were performed after water vapor admission. Significant changes are observed on the spectrum collected after outgassing D_2_O at bt (Figure 6, panel I, curve c). The amide I band at 1640 cm^–1^ changes in shape and increases in intensity. This band has a small NH in-plane bending component and shifts by a few cm^–1^ upon deuteration:^39^ a precise analysis is difficult in a situation where deuterated and nondeuterated NH probably coexist. A more obvious evolution occurs in the region of the amide II mode (mostly a combination of NH in-plane bending and CN stretching). The original band located at about 1528 cm^–1^ is partly but not entirely consumed, while a new band attributable to the amide II′ mode of deuterated peptide linkages appears at 1466 cm^–1^. Moreover, in the ν_NH_ region, only the narrow component at 3307 cm^–1^ is left upon D_2_O vapor admission; the broader component at 3400 cm^–1^ completely disappears. These observations strongly suggest that the amide links in the poly-Gly chains belong to two different populations, one that is susceptible to H/D exchange and another one that is not, being inaccessible and/or stabilized by strong H-bonding. The second explanation sounds more likely since this population gives sharp bands characteristic of well-ordered structures. Both narrow bands in the amide I and in the ν_NH_ regions that resist the H/D exchange are more intense in G/F-AX in comparison with G/AX. This would reflect the higher amount of ordered poly-Gly formed on G/F-AX compared to G/AX. The second derivatives of the spectra (Figure 6, panel II, curve c) seem to indicate that random coils (1642 cm^–1^) are transformed into β-sheets (1617, 1636, 1684, and 1694 cm^–1^) and helices (1650 and 1657 cm^–1^) after H/D exchange while some β-turn initiators (1669 and 1677 cm^–1^) are still present at the surface.

To assess the stability of the self-assembled aggregates formed on the surface, G/F-AX was washed with ultrapure water then dried at rt and outgassed at bt before performing an H/D exchange. IR measurements (Figure 6, panel I, curve d) show that the ν_NH_ band at 3307 cm^–1^ becomes even narrower and is not affected by H/D exchange. Moreover, the peptides bands are still present with significant intensities, and the minima of the amide second derivative spectra (Figure 6, panel II, curve d) are compatible with the presence of β-sheets (1617, 1627, 1633, 1679, 1687, and 1694 cm^–1^) with some helices (1642, 1650, and 1657 cm^–1^) and β-turns (1666 and 1671 cm^–1^) conformations.^39^ This confirms that the formed poly-Gly are in highly packed aggregates anchored on the silica surface by ester bonds, which resist not only hydration and H/D exchange from the gas phase but also washing in the presence of liquid water.^44,45^

The extraction yield of the washing procedure, used to analyze the products with HR-MS, has been evaluated by comparing the integrated areas of the IR spectra of the materials obtained after H/D exchange performed after adsorption of Gly and after subsequent washing with water (curves c and d, respectively), focusing on the 1570–1490 cm^–1^ range, where the signals exclusively due to poly-Gly are present. For G/F-AX, the extraction yield has been estimated to be ca. 24%. In other words, 76% of the nonexchanged amide-containing molecules of the self-assembled structures resist washing and remain chemisorbed on the surface.

It is worth noting here that according to the literature, peptide chains with β-sheet structures are characterized by a high resistance to hydrolysis compared to helical and random-coil conformations. This long lifetime suggests the possibility of acting as stereo-selective templates for further peptide deposition in the emergence of primordial life.^46^

## Conclusions

In comparison to previous studies, the novelty of the present work lies first in a deeper characterization of the successive steps of poly-Gly formation on silica surface. In CVD conditions, Gly seems able to bind covalently to the surface through the formation of ester bonds at the expense of (probably) strained rings, isolated silanols, and nearly free silanol pairs. Esterification of isolated silanols appears irreversible, while NFS seem able to interchange between esterified and free forms and thus to play a special role in surface reactivity. From these ester moieties, longer chains are formed, first in β-turns configurations and later through a process probably involving the ligation of neighboring chains, in longer and more ordered secondary structures including β-sheets. The density of chains can be increased by previous FA treatment.

The observation of secondary structures and their evolution through time are a second important observation with obvious interest for the rise in structural complexity at the origins of life. After a long enough reaction time with gas-phase Gly, a large part of the surface is occupied by highly organized poly-Gly chains that resist desorption and deuterium exchange.

An important question is why this system yields long linear polymers, while many other studies have only reported the formation of the cyclic dimer diketopiperazine (DKP). This will be addressed in a forthcoming publication. As a general conclusion, the complexity of the phenomena observed proves the interest of bringing a surface science approach to the study of the origins of life.

## Supporting Information Available

Results giving additional IR spectra of the silica samples pretreated at different temperatures (rt, 160 °C, with formic acid) under vacuum just before the start of Gly deposition; IR spectra of the F-AX sample after each run of pretreatment in formic acid at 160 °C under vacuum; evolution of the integrated area of amide I, proportional to the concentration of oligomers on the surface, versus the time of Gly deposition by CVD for the three samples G/AX_rt_, G/AX, and G/F-AX along with the corresponding peptide weight loading estimated from TGA measurements; XRD patterns for G/AX_rt_, G/AX, and G/F-AX samples; second derivative in the amide I region of the double difference IR spectra of the intermediate CVD steps from 2.5 to 20 h on G/F-AX; IR spectra of G/AX sample submitted to successive treatments: (a) directly after Gly sublimation for 20 h, (b) after subsequent contact with water v.p. (20 mbar) and outgassing for 30 min at bt, and (c) after H/D exchange and then outgassing of D_2_O for 30 min at bt; and TGA and DTG profiles of the G/F-AX after washing with liquid water at the end of 20 h CVD reaction (PDF)

## Abbreviations

AA: amino acids
bt: beam temperature
CVD: chemical vapor deposition
FA: formic acid
Gly: glycine
HR-MS: high-resolution mass spectrometry
NFS: nearly free silanols
SMA: surface mixed anhydride
