# Supplementary material for: Emergence of Order in Origin-of-Life Scenarios on Mineral Surfaces: Polyglycine Chains on Silica
Source: Langmuir. 2022 Dec 5;38(50):15516–25. doi: 10.1021/acs.langmuir.2c02106 (PMC9776562; doi:10.1021/acs.langmuir.2c02106)
Supplement: Supplementary file 1 — la2c02106_si_001.pdf [file la2c02106_si_001.pdf]

Supporting Information

Emergence of order in origins-of-life scenarios on minerals surfaces: polyglycine chains on silica

Ola El Samrout,[§‡] Marco Fabbiani,[§] Gloria Berlier,[§] Jean-François Lambert,[‡]* and Gianmario Martra[§□]*

[§]*Department of Chemistry, University of Torino, Via P. Giuria 7, 10125 Torino, Italy*

[‡]*Laboratoire de Réactivité de Surface, LRS, Sorbonne Université, Place Jussieu, 75005 Paris, France*

Adsorption and reaction of formic acid on the silica surface at 160 °C

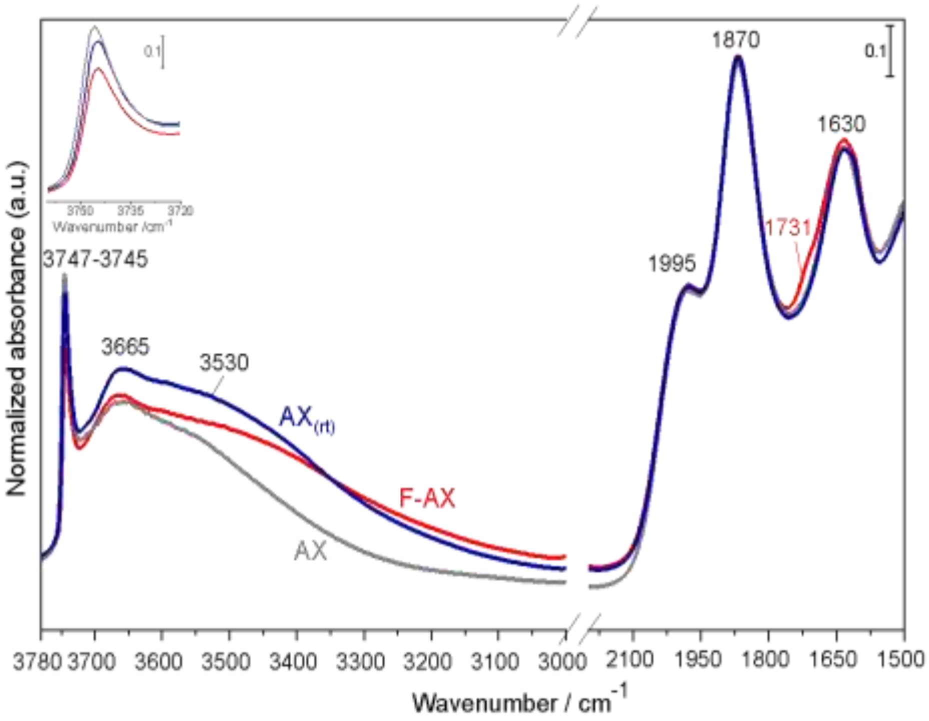

Figure S1. IR spectra of the three samples: $AX_{(rt)}$, AX, and F-AX just before the adsorption of Gly from the vapor phase. The intensity of the spectra has been normalized with respect to the optical thickness ($mg.cm^{-2}$) of the self-supporting pellets prepared for the measurements using the pattern in the 2100-1800 cm^{-1} range.

Discussion of Figure S1

IR spectra under controlled atmosphere were collected for the 3 different silica samples ($AX_{(rt)}$, AX, and F-AX) to check their status just before the start of Gly sublimation. The spectra exhibit a typical pattern with two groups of signals in the range of 3780-3100 cm^{-1} and 2100-1500 cm^{-1}.

Based on available literature data[1,2], the first pattern at high wavenumbers is associated to silanol (Si-OH) stretching νOH as follows: (i) a narrow peak at 3747 cm^{-1}, asymmetric on the low

frequency side, assigned to isolated silanols separated by more than 6 Å, not involved in any intersilanol interaction, (ii) a band in the 3700-3600 cm^{-1} range with a maximum at 3665 cm^{-1} associated to silanols interacting via weak H-bonding (including internal Si-OH not accessible to heavy water), (iii) a broad asymmetric feature from 3600 cm^{-1} down to 3000 cm^{-1} with a maximum at 3530 cm^{-1} assigned to H-bonding silanols located at a distance of 2.5-2.8 Å apart, and which establish mutual strong H-bonded interactions.

A higher dehydration level is reached when the outgassing is performed at 160 °C versus rt. More H-bonding silanol and intraglobular Si-OH are condensed (a certain depletion in the broad band starting from 3700 cm^{-1}) resulting in the formation of more isolated silanols (an increase in the intensity of the narrow band at 3747 cm^{-1}).

The second pattern at low wavenumbers is due to combinations and overtones of symmetric and anti-symmetric bulk modes: $\nu_{sym} + \nu_{as}$ (1995 cm^{-1}), $\nu_{sym} + \nu_{as}$ (1870 cm^{-1}) and 2 ν_{sym} (1630 cm^{-1}). Because they are only due to bulk silica, they were used to normalize the different samples.[1] The IR profile recorded for the F-AX sample after the three runs of pre-treatment in FA shows the persistence of a band at 1731 cm^{-1}. This could indicate to the presence of chemisorbed species on the surface since such species would resist prolonged outgassing. This band was more clearly evidenced by subtracting the spectrum of bare silica, as shown in Figure 2.

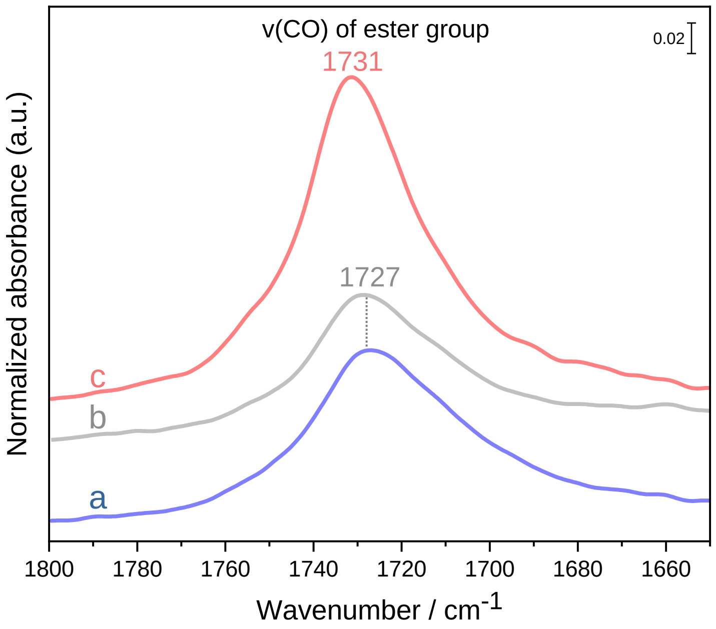

Figure S2. IR spectra of F-AX sample after the (a) 1st, (b) 2nd and (c) 3rd run of pre-treatment in FA where the sample, in each run, was contacted with FA and heated at 160 °C for 2 h, then outgassed at bt. The spectrum of bare SiO_2 obtained after outgassing at 160 °C and subsequent isotopic H/D exchange (by admission of 20 mbar D_2O vapor followed by outgassing at bt) was subtracted as a baseline.

Discussion of Figure S2

IR spectroscopy measurements performed at the end of each run showed the formation of a significant band at around 1727-1731 cm^{-1}, a characteristic band of the presence of ester species on the surface,[3] which increased in intensity after each step.

Gly deposition and polymerization on silica surfaces in CVD conditions

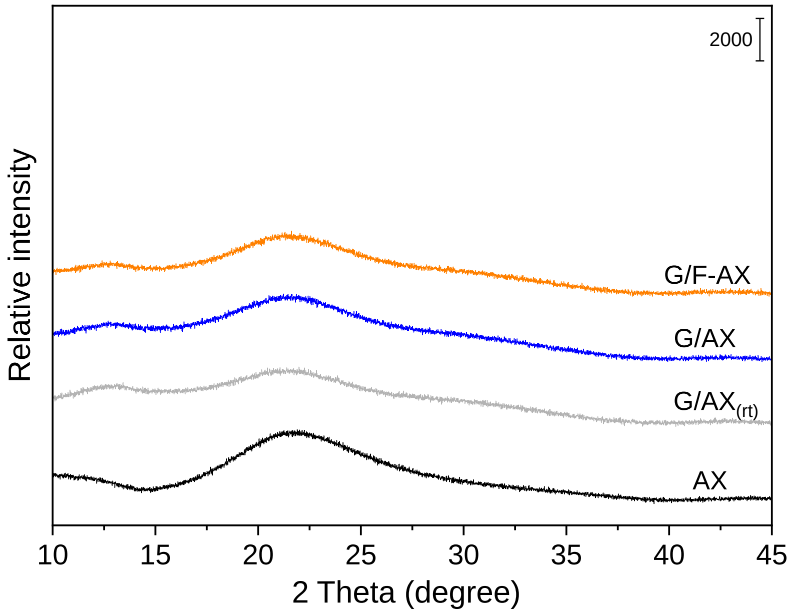

Figure S3. XRD patterns for: AX, bare amorphous silica; G/AX$_{(rt)}$, glycine adsorbed on silica outgassed at rt under vacuum; G/AX, glycine adsorbed on silica outgassed at 160 °C under vacuum; G/F-AX, glycine adsorbed on silica pre-treated with formic acid at 160 °C under vacuum.

Discussion for Figure S3

The XRD patterns of the samples only show the broad background of the amorphous silica support without additional peaks. This confirms the absence of crystalline glycine after deposition by CVD method for 20 h.

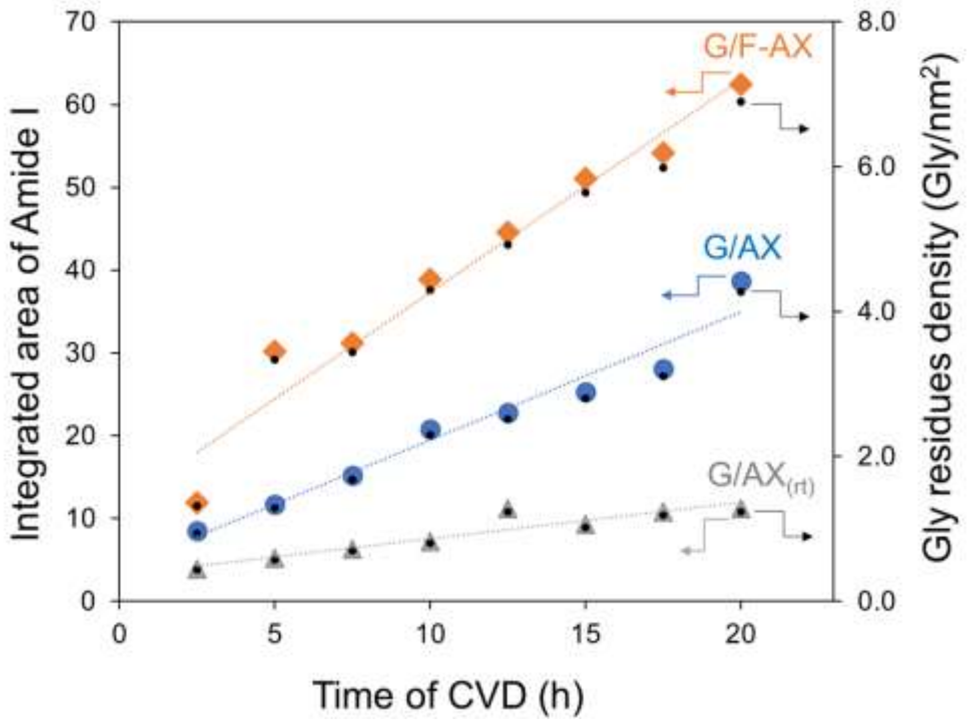

Figure S4. Evolution of the amide I band intensity and Gly residues density (estimated based on TGA measurements) as function of time during Gly deposition by CVD over: G/AX$_{(rt)}$, G/AX and G/F-AX samples.

Discussion of Figure S4

Analysis of the integrated area (proportional to the concentration) of amide I versus time (h) allows to deduce that the Gly polymerization was much more efficient on G/F-AX sample compared to the other samples: higher values of the amide I integrated area were reached in shorter time on G/F-AX. The corresponding Gly residues density for the 3 samples at different CVD time is estimated based on the amount of peptide loading measured on the washed pellet by TGA that implies the amount of peptide loading to be 3.25% by weight after 20 h CVD. This corresponds to 6.9 Gly residues/ nm^2.

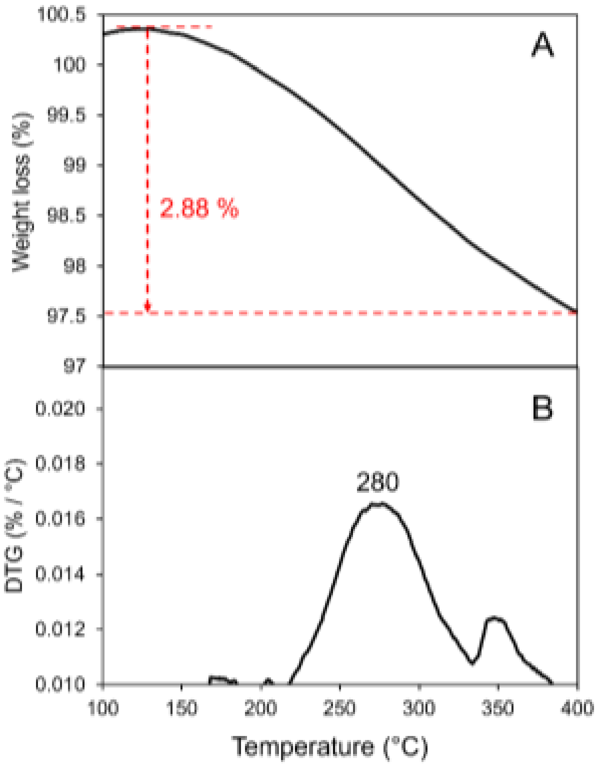

Figure S5. A) TGA and B) DTG profiles of the sample G/F-AX after washing with liquid water at the end of 20 h CVD reaction.

Discussion of Figure S5

The DTG profile recorded for the sample G/F-AX after washing with liquid water at the end of the 20 h CVD reaction shows a major event at around 280 °C. This corresponds to the burning off or destruction of the organic materials remaining on the surface. The weight loss between 130 and 400 °C represents 2.88 % by weight for this sample. When subtracting the weight loss of the corresponding blank silica (0.40 %), the actual peptide loading is estimated to be around 2.48 % by weight on G/F-AX after washing. This value corresponds to around 5.2 Gly residues/nm^2. If washing with liquid water only allows solubilization of ca. 24% of the formed peptides (as stated in the main text, Figure 6) then before washing the peptide weight loading could be concluded to be 6.9 Gly residues/nm^2 after 20 h CVD.

Self-assembly and secondary structures of poly-Gly

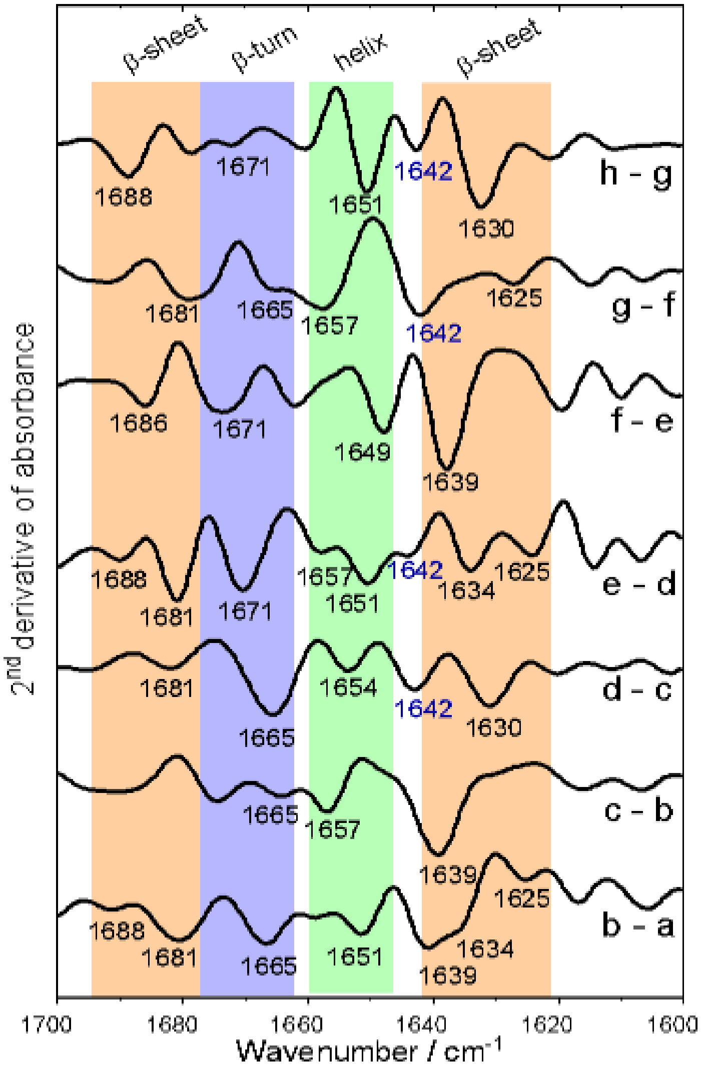

Figure S6. Second derivative in the amide I region of the double difference IR spectra of the intermediate CVD steps from 2.5 h (a) to 20 h (h) on G/F-AX, obtained by subtracting each step from the previous one.

Discussion for Figure S6

As discussed for Figure 5 in the main text, poly-Gly chains of large-scale elements are formed on the surface. The minima of the second derivative of the double difference spectra

represent an additional evidence for the formation of peptides containing β-turn (1665, 1671 cm^{-1}), β-sheet (1625, 1630, 1634, 1639, 1681, 1686, 1688 cm^{-1}), and helical (1649, 1651, 1657 cm^{-1}) conformations[5] starting from 5 h till 20 h CVD on G/F-AX. Some non-ordered chains are also formed as indicated by the minima at around 1642 cm^{-1} for the IR spectra at some intermediate CVD steps.

Effect of hydration/dehydration cycles on grafted poly-Gly

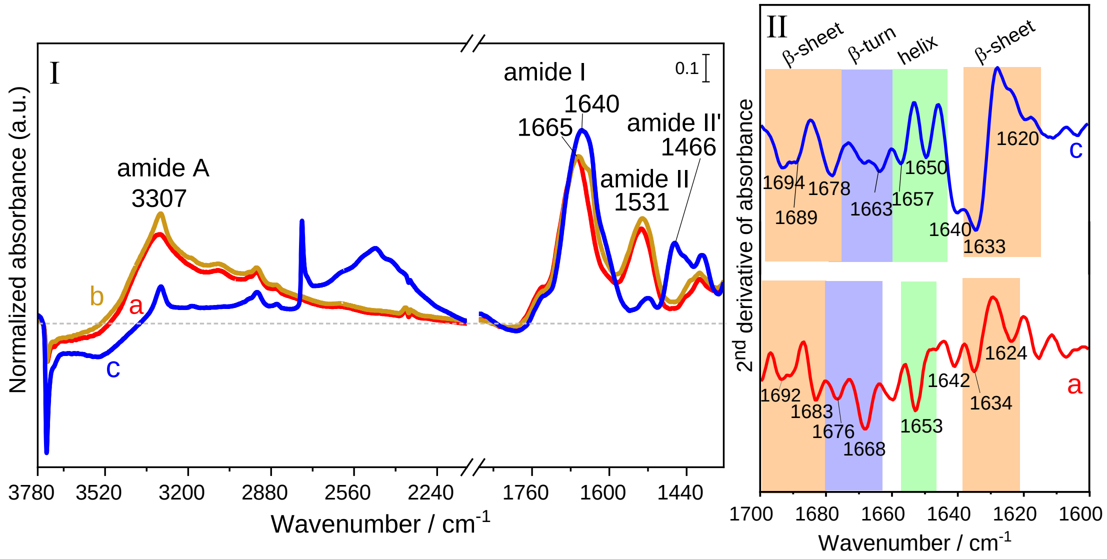

Figure S7. Panel (I): IR spectra of G/AX sample submitted to successive treatments: (a) directly after Gly sublimation for 20 h, (b) after subsequent contact with water v.p.(20 mbar) and outgassing for 30 min at bt, and (c) after H/D exchange and then outgassing of D_2O for 30 min at bt.
The corresponding spectrum of the material obtained before the start of CVD process is subtracted as a baseline. Panel (II) shows the second derivative of the IR spectra a, and c.

Discussion of Figure S7

The general trends observed on G/AX (Figure S7, Panel I) upon water vapor admission are similar to the ones reported for G/F-AX sample (Figure 6 of the main text, Panel I). The second derivative of the spectra obtained after outgassing at bt following the 20 h CVD (Figure S7, Panel

II, curve a) indicates, in coherence with the outcomes reported by Martra at al.[4], that self-assembled structures containing both β-sheet (1692, 1683, 1634, 1624 cm^{-1}) and helical (1653 cm^{-1}) conformations are formed on G/AX sample at the end of the reaction. Some non-ordered structures are also formed on the surface (1642 cm^{-1}).

However, the second derivative of the spectrum obtained after D_2O exchange (Figure S7, Panel II, curve c) revealed, interestingly, that the remaining oligomers were then almost similar to the one obtained on G/F-AX sample directly after CVD (Figure 6 of the main text, Panel II, curve a'): more β-sheet (1694, 1689, 1678, 1633 and 1620 cm^{-1}) and helical (1657, 1650 cm^{-1}) structures are present while a certain amount was still in random coil (1640 cm^{-1}) [6]. Thus, the peptides that resist D-exchange because they are strongly H-bonded resemble those that predominate in the sample with a higher density of adsorbed Poly-Gly.

REFERENCES

(1) Rimola, A.; Fabbiani, M.; Sodupe, M.; Ugliengo, P.; Martra, G. How Does Silica Catalyze the Amide Bond Formation under Dry Conditions? Role of Specific Surface Silanol Pairs. *ACS Catal.* **2018**, *8*, 4558–4568.

(2) Catalano, F.; Alberto, G.; Ivanchenko, P.; Dovbeshko, G.; Martra, G. Effect of Silica Surface Properties on the Formation of Multilayer or Submonolayer Protein Hard Corona: Albumin Adsorption on Pyrolytic and Colloidal SiO 2 Nanoparticles. *J. Phys. Chem. C* **2015**, *119*, 45.

(3) Young, R. P. Infrared Spectroscopic Studies of Adsorption and Catalysis. Part 3. Carboxyllic Acids and Their Derivatives Adsorbed on Silica. *Can. J. Chem.* **1969**, *47* (12), 2237–2247.

(4) Martra, G.; Deiana, C.; Sakhno, Y.; Barberis, I.; Fabbiani, M.; Pazzi, M.; Vincenti, M. The Formation and Self-Assembly of Long Prebiotic Oligomers Produced by the Condensation of Unactivated Amino Acids on Oxide Surfaces. *Angew. Chemie - Int. Ed.* **2014**, *53* (18), 4671–4674.

(5) Barth, A. Infrared Spectroscopy of Proteins. *Biochim. Biophys. Acta - Bioenerg.* **2007**, *1767* (9), 1073–1101.

(6) Cobb, J. S.; Zai-Rose, V.; Correia, J. J.; Janorkar, A. V. FT-IR Spectroscopic Analysis of the Secondary Structures Present during the Desiccation Induced Aggregation of Elastin-

Like Polypeptide on Silica. *ACS Omega* **2020**, *5* (14), 8403–8413.
